# *Bacillus paranthracis* Isolate from Blood of Fatal Ebola Virus Disease Case

**DOI:** 10.3390/pathogens9060475

**Published:** 2020-06-16

**Authors:** M. Jeremiah Matson, Sarah L. Anzick, Friederike Feldmann, Craig A. Martens, Steven K. Drake, Heinz Feldmann, Moses Massaquoi, Daniel S. Chertow, Vincent J. Munster

**Affiliations:** 1 Laboratory of Virology, Rocky Mountain Laboratories, National Institute of Allergy and Infectious Diseases, National Institutes of Health, Hamilton, MT 59840, USA; matthew.matson@nih.gov (M.J.M.); feldmannh@niaid.nih.gov (H.F.); 2 Marshall University Joan C. Edwards School of Medicine, Huntington, WV 25701, USA; 3 Genomics Unit, Rocky Mountain Laboratories, National Institute of Allergy and Infectious Diseases, National Institutes of Health, Hamilton, MT 59840, USA; sanzick@mail.nih.gov (S.L.A.); martensc@niaid.nih.gov (C.A.M.); 4 Rocky Mountain Veterinary Branch, Rocky Mountain Laboratories, National Institute of Allergy and Infectious Diseases, National Institutes of Health, Hamilton, MT 59840, USA; feldmannfe@niaid.nih.gov (F.F.); chertowd@cc.nih.gov (D.S.C.); 5 Critical Care Medicine Department, Clinical Center, National Institutes of Health, Bethesda, MD 20814, USA; steve.drake@nih.gov; 6 Clinton Health Access Initiative, Monrovia, Liberia; mmassaquoi@clintonhealthaccess.org; 7 Laboratory of Immunoregulation, National Institute of Allergy and Infectious Diseases, National Institutes of Health, Bethesda, MD 20814, USA

**Keywords:** Ebola, EBOV, Ebola virus disease, EVD, Africa, West Africa, Liberia, *Bacillus*, *Bacillus cereus*, bacteremia, co-infection, sepsis, hemorrhagic fever

## Abstract

A *Bacillus paranthracis* isolate was cultured from the blood of a fatal Ebola virus disease (EVD) case in Liberia and was identified by whole genome sequencing. Although *B. paranthracis* has only recently been described and is poorly characterized, this case may represent the bacterial co-infection of an EVD patient.

Bacteremia, possibly via gut translocation, might complicate Ebola virus disease (EVD) [1,2,3], and current supportive care guidelines recommend the empiric administration of broad-spectrum antibiotics to all EVD patients [4,5]. However, supporting data are limited as microbiological investigations are not routinely performed in Ebola treatment units (ETUs) during outbreaks. A single case of Gram-negative sepsis was reported in an EVD patient treated in Europe, although the organism was not fully identified, and iatrogenic infection could not be excluded [1]. In one small cohort of 18 EVD patients treated at a military ETU in Sierra Leone, blood cultures were obtained and yielded only one isolate of a coagulase–negative *Staphyloccoci* skin contaminant [6]. Other, indirect evidence of bacteremia in EVD patients hinges on clinical observation [2] and retrospective unbiased deep sequencing [3]. 

Here we report on the microbiological and molecular characterization of a bacterial isolate obtained from the retrospective analysis of a stored whole blood sample obtained from a female patient of unknown age admitted to the ELWA-3 ETU in Monrovia, Liberia on 2 November 2014 [7]. The patient tested positive for Ebola virus by quantitative reverse-transcription polymerase chain reaction (cycle threshold = 23.58) four days following symptom onset. Malaria diagnosis was negative. The patient died two days after admission with no further samples collected. This study was designated “not human subjects research” by the NIH Office of Human Subjects Research Protection (OHSRP) and was approved by the University of Liberia-Pacific Institute for Research and Evaluation (UL-PIRE) institutional review board. 

Upon triage, a whole blood specimen was collected in an EDTA tube and frozen at −80 °C for long-term storage at biosafety level 4 (BSL-4). The gold-standard culture-based approach for the identification of bacteremia was utilized with a lowered input volume due to limited sample availability. A 200 μL aliquot of the whole blood was inoculated into a BD BACTEC Peds Plus /F culture vial with BD fastidious organism supplement and incubated in a BD BACTEC FX40 (Becton Dickinson, https://www.bd.com/). Logarithmic growth by fluorescence was detected 11.5 h after inoculation. The sample was sub-cultured on non-selective sheep blood agar (SBA) and chocolate agar at 35 °C without supplemental CO_2_ and produced heavy monomicrobial growth of dull, gray-white, opaque colonies at 24 h, with weak β-hemolysis evident directly beneath the colonies at 48 h. The isolate was designated RML14492_ELWA-3_3298 (ELWA 3298). 

For identification, the colonies were removed from BSL-4 after inactivation in TRIzol [8] (Thermo Fisher, https://www.thermofisher.com) according to standard operating procedures approved by the Institutional Biosafety Committee, as standard biochemical testing for identification was not readily available in BSL-4. Analysis of nucleic acids by 16S rRNA sequencing and purified protein extract by matrix-assisted laser desorption ionization–time of flight mass spectrometry (MALDI–TOF MS) [9] provided only confident genus-level identification within the *Bacillus cereus sensu lato* group [10]. The lack of strong β-hemolysis on SBA (Table 1) and the geographical proximity of the patient both to human infections of *B. anthracis* and zoonotic infections of *B. cereus* biovar *anthracis* [11] prompted full-genome sequencing for species-level identification, as conventional microbiological testing and other diagnostic assays were not immediately available in BSL-4. A TruSeq DNA Nano kit was used for library preparation and 250 bp paired-end sequencing was performed on a MiSeq sequencer (Illumina, https://www.illumina.com). Raw reads were trimmed, filtered, and coordinate-order sorted using Cutadapt v1.12, FASTX Toolkit v0.0.14, and a custom Perl script. Mira v4.0.2, Velvet v1.2.10, Sequencher (Genecode 5.4.6), and Pilon v1.22 were used for assembly and final polishing. The draft genome de novo assembly resulted in 71 contigs with a total length of 5,681,902 bp, a N50 value of 182,992 bp, and a GC content of 35.2%. This whole genome shotgun project has been deposited at DDBJ/ENA/GenBank under the BioSample identifier SAMN13240933. Following annotation with the NCBI Prokaryotic Genome Annotation Pipeline and average nucleotide identity analysis, the isolate was identified as *B. paranthracis* as it demonstrated a 97.6% identity with 83% coverage to the type strain of *B. paranthracis*, Mn5T. *B. paranthracis* is poorly characterized and was first described in 2017 [12], although current reports describe its isolation from human feces [13] and it may have been responsible for a small emetic and diarrheal outbreak [14]. A limited range of phenotypic testing was performed on the isolate following identification for further characterization (Table 1).

BTyper predicted the ELWA 3298 sequence type (ST) as 1074, based on seven housekeeping genes (*glp*, *gmk*, *ilv*, *pta*, *pur*, *pyc*, and *tpi*) commonly used for *Bacillus cereus sensu lato* multilocus sequence typing (MSLT) [15]. The other currently described isolates of *B. paranthracis* have STs of 26 and 761. MLST phylogenetic analysis, utilizing concatenated sequences of the seven housekeeping genes, was performed. The sequences were aligned with MUSCLE and a maximum-likelihood tree with 1000 bootstraps was generated with PhyML using a general time reversible nucleotide substitution model. The ELWA 3298 isolate fell into clade 1 of the *B. cereus sensu lato* group [15] and a lineage was formed with the other *B. paranthracis* STs, as shown in Figure 1. A MLST minimum spanning tree containing the STs for the known *B. paranthracis* isolates placed ELWA 3298 (ST-1074) closest to the ST-26 *B. paranthracis* isolate, as they share four of seven alleles, as shown in Figure 2. The Mn5T *B. paranthracis* type strain isolate, which has an ST of 761, is more distantly related and is part of the ST-205 clonal complex. Isolates across these STs have been cultured from numerous sources and have been the etiological agents of various human diseases, including sepsis [16].

This is the first report, to our knowledge, that provides species-level identification and phenotypic characterization of a bacterium isolated from the blood of an EVD patient. However, although iatrogenic infection can be ruled out in this patient, as the sample was collected at triage, non-anthracis *Bacillus* spp. are typically regarded as environmental contaminants when isolated from blood [17]. Nevertheless, invasive infections, including sepsis, are increasingly attributed to some non-anthracis *Bacillus* spp., particularly in immunocompromised patients [17]. Given the ability of *Bacillus* spp. to readily colonize the gut, both as transient flora and pathogenically [17], a gastrointestinal source in this patient is plausible if true bacteremia was present [1,2]. With the present limitations, however, caution must be taken with such an interpretation and a determination of the clinical significance of this isolate cannot be definitively made. This finding underpins the need for the continued study of possible bacterial co-infections with EVD. 

## Figures and Tables

**Figure 1:**
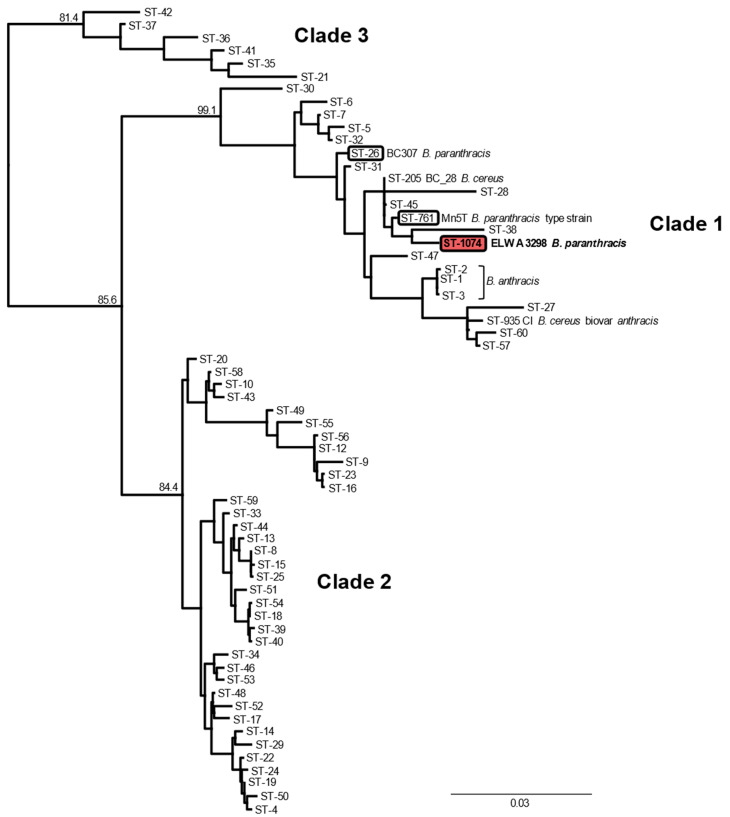
Maximum-likelihood multilocus sequence typing (MLST) phylogenetic tree for the *B. cereus sensu lato* group. Concatenated sequences for seven genes (*glp*, *gmk*, *ilv*, *pta*, *pur*, *pyc*, and *tpi*) were downloaded from the publicly available *B. cereus* database at pubmlst.org. Representative sequence types (STs) for the *B. cereus sensu lato* group were chosen following Priest et al. [15], with additional STs relevant to this report included (1074, 761, 26, 935). The tree was constructed with PhyML using a GTR substitution model and 1000 bootstrap replicates. Support values for the main branches are shown. STs with *B. paranthracis* are indicated with the ST-## enclosed, and the ST for the ELWA 3298 *B. paranthracis* isolate in this report is highlighted red. The tree was rooted with ST-83 *B. pseudomycoides* (not shown). The scale bar represents nucleotide substitutions per site. Bootstrap values are reported as percentages out of 1000 replicates.

**Figure 2:**
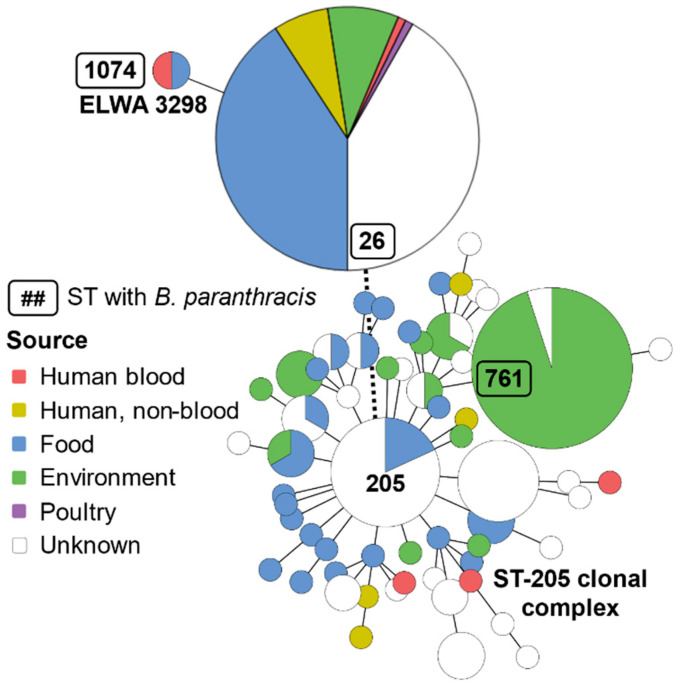
Multilocus sequence typing (MLST) minimum-spanning tree for *B. paranthracis* isolates and related sequence types (STs). STs for *B. cereus sensu lato* isolates were accessed at pubmlst.org and analyzed with GrapeTree. Each node represents a unique ST, and node size is proportional to the number of isolates with that ST. Selected nodes are labelled with their respective STs. Node color indicates the source from which the isolate was obtained. “Human, non-blood” includes vomit, diarrhea, wound swabs, lung tissue, and lung aspirate; “food” includes rice, pasta, food packaging, and other unspecified food; “environment” includes soil, mud, leaves, and rivers. Branch length is logarithmically proportional to the number of allelic differences between the connected STs. The branch with a broken line indicates that ST-26 is not part of the ST-205 clonal complex but still shares multiple alleles.

**Table 1:** Comparison with other *Bacillus* spp.

*Bacillus* Species/Strain	Hemolysis	Motility	Penicillin	Catalase	PEA ^§^ Growth
ELWA 3298, *B. paranthracis*	weak β *	+ ^†^	R ^‡^	+	+
*B. cereus sensu stricto*	strong β	+	R	+	+
*B. cereus* biovar *anthracis*	γ	+/−	S/R	+	unknown
*B. anthracis*	γ	-	S	+	-

Table adapted from American Society for Microbiology’s “Sentinel Level Clinical Laboratory Guidelines for Suspected Agents of Bioterrorism and Emerging Infectious Diseases”, 2017. * β-hemolysis on sheep-blood agar was most evident at 48 h and directly beneath colonies. ^†^ Motility testing was performed with 2,3,5-triphenyltetrazolium chloride (TTC) motility agar using *Escherichia coli* DH10B as a positive control and *Klebsiella pneumoniae* (ATCC 13883) as a negative control. ^‡^ Antimicrobial susceptibility testing was performed with Sensititre broth microdilution (Thermo Fisher, https://www.thermofisher.com) and interpreted according to CLSI M45 MIC criteria; the isolate was also resistant to oxacillin, ampicillin, and cephalosporins.^§^ Phenylethyl alcohol agar.

## Note

This Whole Genome Shotgun project has been deposited at DDBJ/ENA/GenBank under the accession WQMW00000000. The version described in this paper is version WQMW01000000.
